# Observational case series: six neurosurgical patients with septic shock demonstrating clinical improvement after a combination of standard care and blood purification

**DOI:** 10.1186/s40001-021-00614-7

**Published:** 2021-12-20

**Authors:** A. I. Burov, T. A. Abramov, N. S. Kostritca, D. S. Korotkov, G. V. Danilov, Y. V. Strunina, I. A. Savin

**Affiliations:** 1 Federal State Autonomous Institution N. N. Burdenko National Medical Research Center of Neurosurgery of the Ministry of Health of the Russian Federation, Moscow, Russian Federation; 2 Federal State Budget Educational Institution of Higher Education M. V. Lomonosov Moscow State University, Moscow, Russian Federation

**Keywords:** Septic shock, Blood purification, Hemoadsorption, Inflammatory mediators adsorber, High adsorption capacity membrane, Continuous renal replacement therapy

## Abstract

**Background:**

For patients with primary brain injury, septic shock is especially dangerous due to the possibility of secondary cerebral damage. The key factor of sepsis-associated brain injury is inflammatory mediators, pathogen and damage-associated molecular patterns (PAMPs, DAMPs) release. Theoretically, blood purification may be beneficial for patients with primary brain injury due to its possibility for fast removal of inflammatory mediators.

**Case presentation:**

We report on six post-neurosurgery septic shock patients treated with combined blood purification (CBP), which included CRRT with high adsorption capacity membrane in combination with CytoSorb adsorber. Clinical improvement in the course of CBP was registered in all patients. Three patients had a stable clinical improvement; the other three patients had only a transient improvement due to underlying neurological and cardiac deficits aggravation. We observed septic shock reversal in four patients. The key observations of the case series are a significant decrease in MOF severity (measured by SOFA score) and in catecholamine need (not statistically significant). By the end of CBP we observed a significant decrease in blood lactate, PCT and IL-6 levels. Two patients demonstrated level of consciousness increase in the setting of CBP therapy measured by GCS and FOUR score.

**Conclusion:**

This case series demonstrates that CBP therapy may have a role for septic shock patients with primary brain injury.

## Introduction

It has been described that septic shock is associated with a higher risk of mortality in ICU patients [1]. It is important to be mindful of septic shock being the driver of the secondary brain damage in neurosurgical patients. The mechanisms of pathogen and damage-associated molecular patterns (PAMPs, DAMPs) based immunity activation, cytokine release-driven endothelial damage and subsequent hematoencephalic barrier function affliction are well described [2]. The described phenomena lead to vasogenic brain edema development [3, 4]. Cerebral perfusion decreases in septic shock, being the secondary brain injury driver, is especially deleterious to patients with pre-existing cerebral blood flow autoregulation disturbances [2].

Even though the benefits of combined antibacterial therapy in septic shock have been demonstrated [1], in some cases its timely use does not entail fast hemodynamic stabilization and multiple organ failure (MOF) regress. For patients not responding to standard therapy, blood purification techniques may potentially be effective. The most important reason for blood purification in neurosurgical patients is the maximally fast PAMPs and DAMPs elimination, allowing to minimize secondary brain damage [3].

The use of CRRT with standard membranes in continuous veno-venous hemofiltration mode was not associated with mortality decrease [5]. Apart from that, conventional CRRT has not proved effective in terms of cytokine elimination, whereas cytokines are considered key factors of hemodynamic instability progression and MOF development [5]. Nowadays there exist other adjuvant methods, providing for fast and effective cytokine, PAMPs and DAMPs elimination from systemic blood flow: increased adsorption capacity membranes, inflammatory mediators adsorbers and lipopolysaccharide columns [6–8]. Several studies on CytoSorb (Cytosorbents, USA) demonstrated effective elimination of substances, described above [9–12].

To our knowledge, publications on the use of adjuvant blood purification methods in neurosurgical patients with septic shock are scarce. This case series describes the treatment of six patients after neurosurgical interventions who developed septic shock and received adjuvant therapy with combined blood purification.

## Methods

We report on six adult post-neurosurgery septic shock patients with median age of 62 [55–71] (CH, G, K, M, P, S). In all of them septic shock was diagnosed according to Sepsis-3 criteria [2]. Patient characteristics are presented in Table 1. All patients received standard therapy according to Surviving Sepsis Campaign guidelines [1]. Antibacterial therapy was started within 1 h of septic shock diagnosis. Dosages of antibacterial drugs were used in accordance with the recommendations for patients on CRRT; therapeutic drug monitoring was not available at our facility at the time. All patients were non-responders to standard therapy within 6 or more hours of septic shock: vasopressor demand continued to rise (norepinephrine (NE) > 0.1 µg/kg/min or a combination of vasopressors) and MOF was progressing (SOFA score ≥ 11). The median SOFA score was 14 [12;16]. All patients had AKI (KDIGO I-III).

**Table 1:** Patient characteristics, major medical history, antibiotic therapy, scoring pre- and post-treatment

Case no.	Name	Sex	Age	Major pathology	Source of infection	Pathogen	Antibiotics	Time before CBP hours	Initial SOFA score	Initial GCS	Initial FOUR
1	S	F	71	Brain tumor removal	Urinary tract infection	Not determined	Meropenem, linezolid, ciprofloxacin	10	12	10	11
2	G	F	82	Aneurysm clipping after SAH	Pneumonia	*Klebsiella pneumoniae*	Meropenem, linezolid, fluconazole, polymyxin E	20	12	6	8
3	Ch	M	55	Brain trauma	Bloodstream infection	*Staphylococcus epidermidis*	Meropenem, tigecycline, polymyxin E	10	11	6	8
4	P	M	29	Aneurysm clipping after SAH	Bloodstream infection	*Escherichia coli*	Meropenem, linezolid, fluconazole, polymyxin E	6	16	4	5
5	K	M	56	Aneurysm clipping after SAH	Bloodstream infection	*Escherichia coli*, *Morganella morganii*	Meropenem, linezolid, polymyxin E	20	16	4	2
6	M	M	68	Brain tumor removal, PE, post-CPR	Pneumonia	MSSA	Meropenem, linezolid	6	18	10	13

CBP consisted of CRRT and inflammatory mediators’ adsorber (CytoSorb) in all patients. CBP therapy was started within 10 h after septic shock diagnosis in 4 patients and within 20 h in the other 2 patients.

CRRT was performed in CVVHDF mode using Prismaflex (Gambro Medical) with AN69ST set in 5 patients (CH, G, M, P, S) and lipopolysaccharide elimination membrane (oXiris (Baxter International Inc.) in 1 patient (K) who had Endotoxin Activity Assay (EAA) test 0.67. CBP was performed with CytoSorb adsorber in all our patients. CytoSorb was placed in the post-filter position for 24 h for all patients. Blood flow rate was 150–200 ml/min. Anticoagulation was performed with heparin (5–15 U/kg/h) in 2 patients, monitored by aPTT with target level 1.5–2 times higher than the normal; citrate in 3 patients; 1 patient was not anticoagulated due to severe thrombocytopenia. The overall CBP therapy duration was 24 h for all patients. In four patients, CRRT and inflammatory mediators adsorption were started simultaneously in combination. Patient K received CRRT with AN69 membrane in CVVHDF mode for 20 h, patient M—for 72 h before CytoSorb installation.

To assess CBP effects, vasopressor requirements, SOFA score and lactate levels were recorded in all patients (Table 2). Shock reversal was recorded, defined as vasopressor requirement decrease and lactate level normalization. Procalcitonin (PCT), C-reactive protein (CRP) and IL-6 levels were evaluated (Table 3). Glasgow Coma Scale (GCS) and FOUR score were calculated to assess CBP effects on the consciousness level. We assessed outcome in terms of days on CRRT, 7-, 28-day and hospital mortality (Table 4).

**Table 2:** Patient clinical parameters, scoring pre- and post-treatment

Name	NE Before CBP µg/kg/min	NE 24 h µg/kg/min	NE 48 h µg/kg/min	NE 72 h µg/kg/min	Lac Before CBP mmol/l	Lac 24 h mmol/l	Lac 48 h mmol/l	Lac 72 h mmol/l	SOFA score before CBP	SOFA score 24 h	SOFA score 48 h	SOFA score 72 h
S	0.77	0.09	0	0	4.40	2.2	4.7	4.7	12	8	6	8
G	1	0	0	0	2	1.8	2.9	1.8	12	4	5	4
Ch	0.18	0.23	0.17	0.16	2.1	1.9	1.3	0.7	11	8	9	9
P	2.13	1.64	1.8	3.6	9.8	7.3	7.7	5.8	16	15	15	18
K	0.71	0.15	0.15	1	2	1.1	1.2	1.5	16	14	15	15
M	1.68	2.24	–	–	9.6	7.9	–	–	18	17	–	–

**Table 3:** Inflammatory mediator’s levels pre- and post-treatment

Name	PCT Before CBP ng/ml	PCT 6 h ng/ml	PCT 12 h ng/ml	PCT 24 h ng/ml	Cytokines Before CBP pg/ml		Cytokines 6 h pg/ml		Cytokines 24 h pg/ml		CRP Before CBP mg/l	CRP 24 h mg/l	CRP 48 h mg/l	CRP 72 h mg/l
					IL-6	IL-10	IL-6	IL-10	IL-6	IL-10				
S	> 200	> 200	81.74	86.62	3797	585	464	31	85	21.1	34.7	23.7	35.4	28.2
G	18.13	4.15	4.46	4.43	194	9,5	34	5.0	76	5.7	71.6	48.6	57.2	48.8
Ch	> 200	46.72	34.59	22.52	56	< 5.0	34	< 5.0	24	< 5.0	182	181.5	102.2	69.8
P	139.53	57.65	66,53	78.29	32,280	1030	9142	80.6	6352	104	215.8	250	254.4	298.1
K	56	37.8	32	29.34	141	71.8	19.46	19.3	20.98	9.8	257.3	167.2	131.8	68
M	5.32	1.79	1.65	1.43	1598	133	592	50.1	344.6	14.8	95.1	129.7	–	–

**Table 4:** Patient scoring pre- and post-treatment and patient outcome

Name	Initial GCS	GCS 24 h	GCS 72 h	Initial FOUR	FOUR 24 h	FOUR 72 h	CRRT days	Shock reversal	7-day mortality	28-day mortality	Hospital mortality
S	10	10	11	11	11	13	0	Yes	No	No	No
G	6	6	9	8	8	10	0	Yes	No	Yes	Yes
Ch	6	6	6	8	8	8	1	Yes	No	No	No
P	4	4	3	5	5	0	2	No	Yes	Yes	Yes
K	4	4	3	2	2	0	2	Yes	Yes	Yes	Yes
M	10	10	–	13	13	–	0	No	Yes	Yes	Yes

Statistical data analysis was carried out using the R environment (version 3.6.1) in IDE R Studio (version 1.2.1335). Statistical hypotheses about the difference in the distribution of quantitative variables in the samples were tested using the nonparametric Wilcoxon test. The results of testing hypotheses were considered statistically significant at a level of *p* < 0.05.

## Results

Clinical improvement during the course of CBP was registered in all our patients. 3 patients (CH, G, S) had a stable clinical improvement (MOF regress and lactate level decrease); the other 3 patients (P, K, M) had a transient improvement (vasopressor dose decrease (P, K), SOFA score and lactate level reduction (P, K, M)). We observed septic shock reversal in four patients (CH, G, K, S). For those with transient improvement, it was conditioned by their malignant edema and following cerebral herniation in the setting of the underlying condition (P, K) or cardiac insufficiency progress after massive pulmonary embolism (M).

**Fig. 1:**
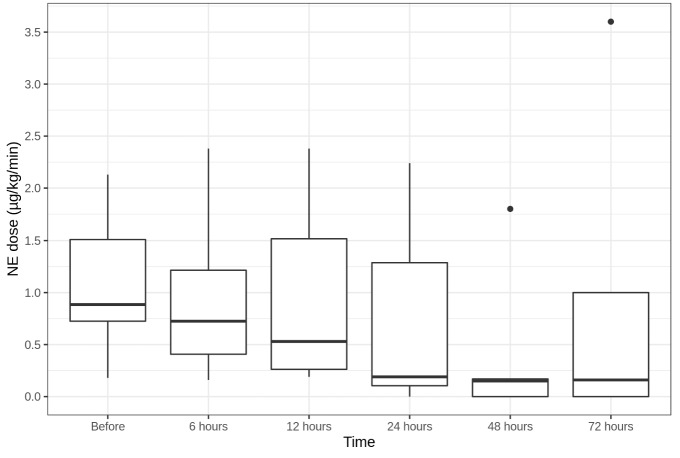
NE dose dynamics (µg/kg/min)

**Fig. 2:**
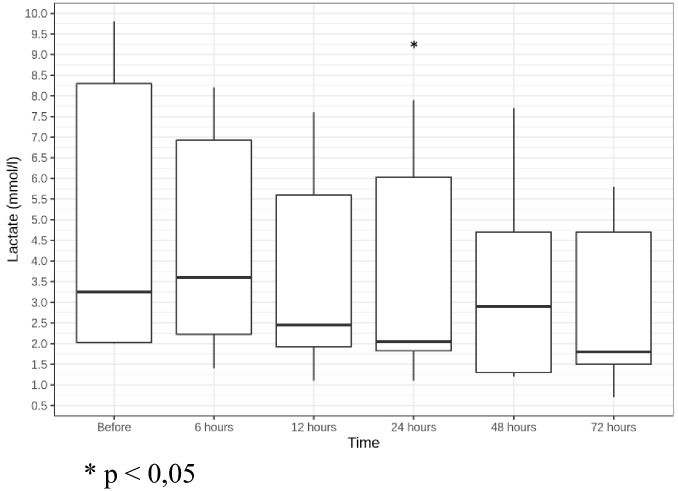
Lactate level dynamics (mmol/l). *p < 0.05

Median NE demand for all patients reduced from a median of 0.89 [0.73;1.51] µg/kg/min before CBP to 0.19 [0.11;1.29] µg/kg/min in 24 h and to 0.16 [0;1] µg/kg/min in 72 h after CBP initiation (not statistically significant) (Fig. 1). Hemodynamic stabilization was accompanied by a decrease in blood lactate from a median of 3.25 [2.03;8.3] mmol/l before CBP to 2.05 [1.83;6.03] mmol/l in 24 h (*p* = 0.031) and to 1.8 [1.5;4.7] mmol/l in 72 h after CBP initiation (not statistically significant) (Fig. 2). Two patients (G, S) demonstrated quick vasopressor demand reduction; vasopressor support could be discontinued for them 48 h after CBP initiation.

**Fig. 3:**
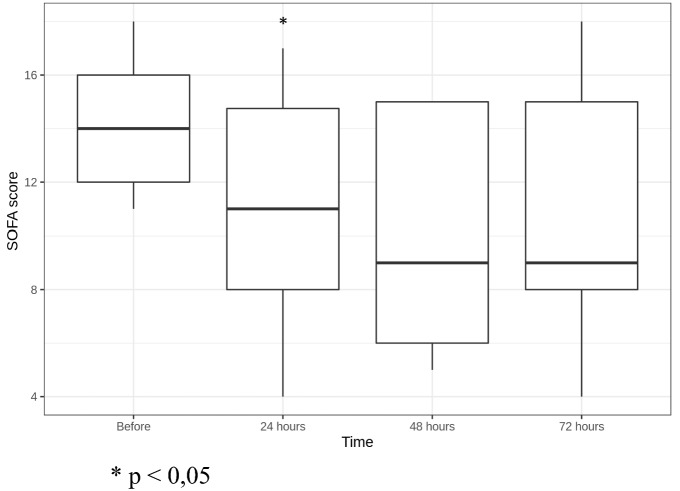
SOFA score dynamics. *p < 0.05

Mean SOFA score decreased from a median of 14 [12;16] before CBP to 11 [8;14.75] in 24 h (*p* = 0.036) and to 9 [8;15] in 72 h after CBP initiation (not statistically significant) (Fig. 3). SOFA score changes were conditioned by renal (G, CH, S, P, K), cardiovascular (G, S) and hepatic (CH, K) insufficiency regress, lung function restoration (G, M, S) and consciousness level rise (G, S). At the same time, in patients G and S thrombocytopenia progressed due to disseminated intravascular coagulation (DIC) development in the setting of septic shock (platelet concentration decreased from 179 to 70*10^9^/l (G) and from 80 to 21*10^9^/l (S) in 24 h after CBP initiation); this conditioned some moderate SOFA score increase.

**Fig. 4:**
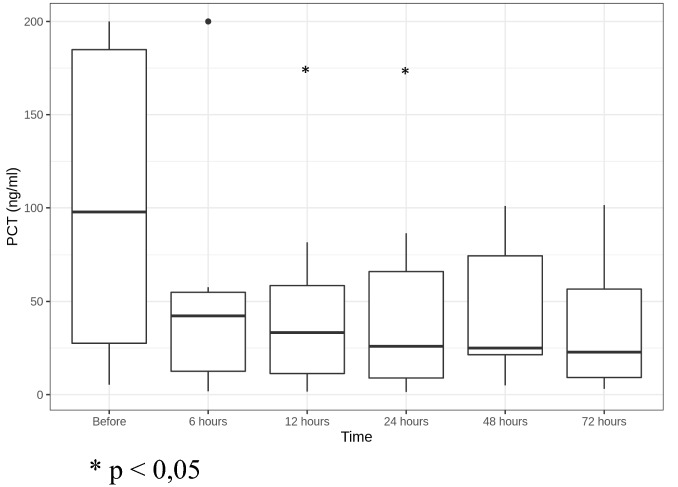
PCT level dynamics (ng/ml). *p < 0.05

**Fig. 5:**
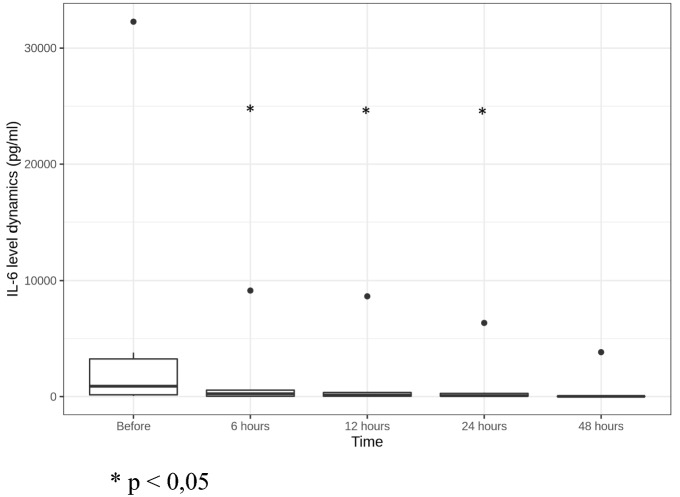
IL-6 level dynamics (pg/ml) *p < 0.05

We registered fast statistically significant decrease of PCT and IL-6 levels: PCT reduced more than 66% in 12 h and more than 74% in 24 h after CBP initiation (Fig. 4); IL-6—more than 71% in 6 h and 95% in 24 h after CBP initiation (Fig. 5).

**Fig. 6:**
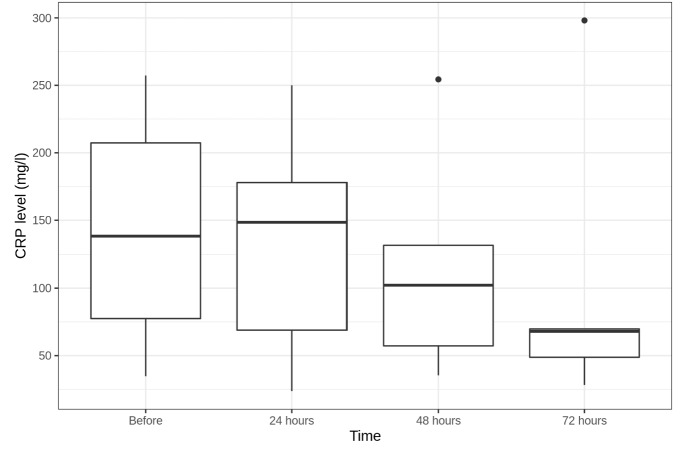
CRP level dynamics (mg/l)

Median CRP level decreased from 138.55 [77.48;207.35] mg/l before CBP to 102.2 [57.2;131.8] mg/l in 48 h and to 68 [48.8;69.8] mg/l in 72 h after CBP initiation (both—not statistically significant) (Fig. 6).

Two patients demonstrated improved level of consciousness during CBP therapy: S (GCS from 10 to 11; FOUR score from 11 to 13) and G (GCS from 6 to 9; FOUR score from 8 to 10).

There were no adverse events during the procedure.

## Discussion

Combined blood purification is becoming widely used in contemporary ICUs, but there are very few reports on the use of any blood purification methods in neurosurgical ICU [13].

In this case series, six patients with septic shock (median SOFA score 14) and AKI were treated with CBP therapy. The treatment was associated with a significant reduction of SOFA score, lactate and PCT levels at 24 h after CBP initiation. 83% of patients (5/6) demonstrated vasopressor demand decrease within 6–48 h of CBP start. Shock reversal was observed in four patients. Stable clinical improvement was noted in 50% (3 out of 6) of patients already within 24 h and then continued at 72 h after CBP initiation. We observed similar data in other publications concerning general ICU patients [14–16]. One patient (G) with stable clinical improvement died 25 days after CBP start due to cerebral hemorrhage; however, treatment of septic shock was successful. Other two patients with stable clinical improvement were discharged to the rehabilitation center.

The reason for only transient improvement of the other 50% of patients is related to their underlying condition (brain herniation and brain death (P, K) or massive pulmonary embolism (M)). The improvement was evident, though, and one of these patients even had a shock reversal. We think that the high early mortality (50%) in our case series is explained primarily by the specifics of severe cerebral injury. In addition, MOF severity by SOFA score in our patients was higher than in most studies on septic shock.

Thrombocytopenia progress in G and S was conditioned by DIC development in the setting of septic shock. In our opinion, this phenomenon was not the adverse effect of the CBP treatment, but rather a characteristic feature of a typical DIC syndrome progression, when platelet concentration dramatically decreases [6, 17].

Studies show that the degree of hypercytokinemia correlates with mortality [7], and that the highest mortality rate is observed in patients with simultaneously increased pro- and anti-inflammatory cytokine levels [8]. In our case series, three patients (M, P, S) had excessive levels of both types of cytokines parallel to high vasopressor demand, lactate level and SOFA score. Despite their severe condition, we observed clinical improvement in the course of CBP (transient or stable).

Cytokine levels dynamics characterize both septic shock treatment and CBP effectiveness [11]. Gruda et al. stated that 5 h of cytokine adsorption could reduce IL-6 level by 91 ± 3.0% [10]. In the described case series, cytokines elimination was observed during the whole procedure (24 h) with the maximum effect during the first 6–12 h. Cytokine clearance was more prominent in patients with extremely high (> 1000 pcg/ml) initial IL-6 levels (M, P, S)—more than 78% in 24 h. Very similar data on direct proportion of cytokines elimination to their initial concentrations were described in other publications [18].

Of special interest is the PCT in the setting of antibacterial therapy and its dynamics during blood purification. Substantial PCT reduction could indicate effective antibacterial therapy and adequate infection control in general; PCT dynamics also can be used as a prognostic marker. According to the literature, a twofold decrease in the PCT level in blood within 5 days is associated with a favorable outcome in patients with sepsis. The mortality rate of patients with a 50% or more decrease in PCT was 29%, and out of those with a decrease of less than 50% the mortality reached 86% [19]. We observed a significantly greater decrease in the PCT level during the blood purification procedure. It has to be noted that PCT is eliminated by the adsorber and some other blood purification methods [20]. PCT reduced significantly in all patients at 6–12 h and then continued to decrease at 18 h of CBP at lower speeds (except for Patient P). The most pronounced PCT elimination (about twofold or more) took place within the first 6 h. After CBP was discontinued, two patients (G, S) demonstrated a small transient PCT increase, then again a decrease at 72 h. In our opinion, unusual PCT dynamics in patient P (the concentration decreased by 59% at 6 h, and started to rise at 12 h, the increase continued until patient’s death) could be explained by underperformance of antibacterial therapy or non-controlled infection source presence.

Matsui et al. reported that CRRT use in patients with stroke had a beneficial effect on the consciousness level [13]. Based on the improved level of consciousness (GCS and FOUR scale increase) parallel to shock reversal in two patients (G, S), we hypothesize on the positive CBP influence on the neurological status in comparison with the standard septic shock therapy. We assessed the change in neurological status primarily using the FOUR scale, taking into account its advantages in patients on mechanical ventilation. The FOUR score evaluation made it possible to assess the changes in neurological status accurately and quickly, which was especially important for our patients.

There were no obvious CBP therapy-related complications.

This case series has some limitations: a small number of patients of various ages and different pathologies. The clinical outcome in neurosurgical patients is often bound to the main disease. In this case series, three patients had terminal episodes due to underlying neurological and cardiac conditions; these explain the lack of statistically significant results at 48 and 72 h. We used two different types of CRRT membranes.

## Conclusion

The most important finding of the current case series describing patients admitted with primary brain injury who developed septic shock, is that we observed shock reversal in four patients within 2 days. We hypothesize that this was the result of complex intensive care, which included basic septic shock treatment and CBP therapy. Our results suggest that CBP therapy may have a role for septic shock patients after neurosurgical interventions, which should be tested in prospective randomized controlled trials in this specific population.

## Data Availability

The data have been collected and stored according to the personal data and health information storage regulations and are available upon request to ensure the case report data transparency.

## Abbreviations

AKI: Acute kidney injury
aPTT: Activated partial thromboplastin time
CBP: Combined blood purification
CRRT: Continuous renal replacement therapy
CPR: Cardio-pulmonary resuscitation
CVVHDF: Continuous veno-venous hemodiafiltration
DAMP: Damage-associated molecular pattern
DIC: Disseminated intravascular coagulation
EAA: Endotoxin activity assay
GCS: Glasgow Coma Scale
MSSA: Methicillin-sensitive *Staphylococcus aureus*
MOF: Multiple organ failure
PAMP: Pathogen-associated molecular pattern
PE: Pulmonary embolism
SOFA: Sequential organ failure assessment score
KDIGO: Kidney Disease Improving Global Outcomes
